# Paulownia Leaves as A New Feed Resource: Chemical Composition and Effects on Growth, Carcasses, Digestibility, Blood Biochemistry, and Intestinal Bacterial Populations of Growing Rabbits

**DOI:** 10.3390/ani9030095

**Published:** 2019-03-18

**Authors:** Adham A. Al-Sagheer, Mohamed E. Abd El-Hack, Mahmoud Alagawany, Mohammed A. Naiel, Samir A. Mahgoub, Mohamed M. Badr, Elsayed O. S. Hussein, Abdullah N. Alowaimer, Ayman A. Swelum

**Affiliations:** 1 Animal Production Department, Faculty of Agriculture, Zagazig University, Zagazig 44511, Egypt; mohammednaiel.1984@gmail.com; 2 Poultry Department, Faculty of Agriculture, Zagazig University, Zagazig 44511, Egypt; dr.mohamed.e.abdalhaq@gmail.com (M.E.A.E.-H.); dr.mahmoud.alagwany@gmail.com (M.A.); 3 Department of Microbiology, Faculty of Agriculture, Zagazig University, Zagazig 44511, Egypt; samir_mahgoub@yahoo.com; 4 Agricultural Engineering Department, Agriculture Faculty, Zagazig University, Zagazig 44511, Egypt; mbadr757@yahoo.com; 5 Department of Animal Production, College of Food and Agriculture Sciences, King Saud University, P.O. Box 2460, Riyadh 11451, Saudi Arabia; shessin@ksu.edu.sa (E.O.S.H.); aowaimer@ksu.edu.sa (A.N.A.); 6 Department of Theriogenology, Faculty of Veterinary Medicine, Zagazig University, Zagazig 44511, Egypt

**Keywords:** growing rabbits, intestinal microbiota, nutrient digestibility, paulownia tree leaves, *Paulownia tomentosa*

## Abstract

**Simple Summary:**

Paulownia trees are grown as a woody biofuel crop, and they yield large amounts of leafy biomass rich in nitrogen. However, there is limited information on the use of paulownia leaves as an animal feed resource. Hence, this study was conducted to assess the effects of paulownia leaf meal (0%, 15%, and 30% in diets) on the growth performance, nutrient digestibility, blood biochemistry, and intestinal microbiota of growing rabbits. The chemical analyses of paulownia leaves indicated that most of the nutrients are similar to those in alfalfa hay. The in vivo results showed that the use of up to 15% paulownia leaf meal instead of alfalfa hay in the diets of the rabbits did not have any negative effects on their performance, nutrient digestibility, and blood constituents. In addition, a notable reduction in both cecal and feed pathogenic bacteria was observed when paulownia leaf meal was included in the diets.

**Abstract:**

This experiment was conducted to study the effects of paulownia leaf meal (PLM) as a nontraditional feed on the growth, carcasses, digestibility, blood chemistry, and intestinal microbiota of growing rabbits. Sixty rabbits (5-weeks old) were randomly allotted to three dietary treatments containing three amounts of PLM (0%, 15%, and 30%). The results showed that PLM has a higher content of ether extract, organic matter, methionine, tyrosine, histidine, manganese, and zinc than alfalfa hay. Body weight gain decreased when 30% PLM was provided. The best feed conversion ratio was recorded in the rabbits fed 15% PLM. A notable increase in high-density lipoprotein levels with a significant decrease in low-density lipoprotein was noted in the rabbits fed the PLM diets. Total fungi and Enterobacteriaceae and total bacterial count in the feed were significantly reduced because of PLM. In the cecum, coliforms, Enterobacteriaceae species, and total bacterial count declined in the rabbits fed the PLM diets. Conclusively, up to 15% PLM can be used in rabbit diets without any deleterious effects on the performance, nutrient digestibility, and blood constituents. In addition, dietary inclusion of PLM has the potential to reduce cecal pathogenic bacteria in rabbits.

## 1. Introduction

In many countries, including Egypt, the animal feed industry is facing major challenges because of high prices of energy and protein sources such as maize and soybean meal, both of which are mainly used in animal feeds [1,2]. Moreover, the low quality and quantity of animal feeds are serious problems that limit the productivity of livestock worldwide. The importance of shrubs, trees, and herbs as fodder sources has been suggested because of their nutritional value (when compared with fodder trees, herbs, grasses, and shrubs with relatively higher levels of neutral detergent fiber, minerals, and crude protein) for grazing animals in low-quality grazing areas for long periods of the year [3,4]. Nowadays, nutritional solutions and strategies are being used to resolve these crises by using alternative feed ingredients, such as tropical and subtropical plants in rabbit rations [5]. Rabbits have become an essential source of meat, and rabbit production is recommended in many countries with meat shortages. In addition, rabbits have advantages such as the best growth and productivity to overcome protein deficiency [6].

Paulownia trees are grown for their wood, which is used to make furniture, musical instruments, and flooring. Paulownia trees are produced in some countries, such as Bulgaria, and because of their good nutritional value, they can be used as an alternative feed ingredient. The leaves of paulownia could be used as an alternative feed ingredient for different animals because of their varied biochemical properties [7]. According to El-Showk and El-Showk [8], paulownia leaves are rich in minerals such as calcium (2.1%), phosphorus (0.6%), zinc (0.9%), and iron (0.6%). Koleva et al. [9] reported that paulownia leaves contain 15.1% cellulose and 8.8% crude protein. Koleva et al. [10] suggested that paulownia leaves can be used as a feed ingredient for some monogastric animals and ruminants. 

However, to date, there is limited information on the optimal use of paulownia leaves as an animal feed resource to overcome the shortage of protein sources for livestock animals. In this study, paulownia leaf meal (PLM) was used as a novel feedstuff to replace alfalfa hay as a high-quality forage in the diet of growing rabbits. Thus, the objective of this study was to analyze the effects of PLM as a nontraditional feed on the growth, carcasses, nutrient digestibility, blood chemistry, and intestinal microbiota of rabbits.

## 2. Materials and Methods

This experiment was performed at the Rabbit Research Farm, Animal Production Department, Agriculture College, Zagazig University, Egypt. All the protocols were performed according to the experimentation guidelines of the Ethics of Animal Use in Research Committee of Zagazig University (approval number: ZU-IACUC/2/F/94/2018).

### 2.1. Source and Preparation of Paulownia Leaves

Paulownia (*Paulownia tomentosa*) leaves were harvested and collected in October 2016. They were collected from 100 six-month-old trees in an orchard located in Bani-Salama Village, Wadi El-Natroun in Beheira Governorate, Egypt (30° 26′N 30° 14′E). The orchard has an area of two acres containing 2500 trees. About 4 kg of fresh leaves was collected from each tree. The average moisture content was 80.22%. The paulownia leaves were transported to the lab of the Animal Production Department, Agriculture College, Zagazig University for further processing. The leaves were dried, ground, and stored in airtight bags until further analysis (proximate and amino acid profile) and use. The paulownia leaves were ground using a grinding machine designed at the Department of Agricultural Engineering, Faculty of Agriculture, Zagazig University for producing a nontraditional feed for growing rabbits to improve product quality. The highest values of machine productivity and overall machine efficiency were 435 kg/h and 90.60%, respectively. The optimum operating parameters of the milling machine were 1600 rpm drum speed by using a 450 kg/h feed rate of PLM.

### 2.2. Proximate and Amino Acid Analyses of Paulownia Leaves

The chemical composition of PLM, alfalfa hay, and experimental diets were estimated using Association of Official Analytical Chemists (AOAC) methods [11]. All samples were analyzed in triplicate for dry matter (DM), crude protein (CP), ether extract (EE), crude fiber (CF), and ash (methods 930.15, 954.01, 920.39, 978.10, and 942.05, respectively). Neutral detergent fiber (NDF) was also determined [12]. Nitrogen-free extract (NFE) was calculated using the following equation:

NFE (%) = 100 − (CP (%) + EE (%) + ash (%) + CF (%)).

The mineral contents (method 984.27) were analyzed using the atomic absorption spectrophotometer in the Central Laboratory for Food and Feed analysis (CLFF), Agriculture Research Center, Ministry of Agriculture, Cairo, Egypt. Amino acid analyses of PLM and experimental diets were performed using an automated amino acid analyzer after hydrolyzing the samples of paulownia leaves and diets with 6 M HCl at 110 °C for 24 h, according to Ogunbusola et al. [13], and the sulfur amino acids were estimated using the method of Bassler and Buchholz [14]. Tryptophan was not estimated because it is demolished through acid hydrolysis. Also, during sample preparation, glutamine is converted to glutamate and asparagine to aspartate, so the values mentioned for these amino acids (Glx and Asx) represent the sum of acid and amide.

### 2.3. Animal, Design, and Alimentation

Sixty New Zealand White rabbits (male, 5-weeks old) were weighed and randomly allotted to three dietary treatments containing three amounts of paulownia powder (0%, 15%, and 30%) for 8 weeks. Each treatment involved 10 cages, where two animals were maintained in 1 cage. The rabbits were housed in the cages (30 cm width × 50 cm length × 40 cm height) and reared under the same managerial conditions, and they were fed to achieve their requirements, according to De Blas and Mateos [15]. All diets were formulated, pelleted, and stored in the farm during the study (Table 1). The ingredient and diet analyses were performed according to AOAC [11].

### 2.4. Growth Performance and Carcass Traits

Feed intake (FI), body weight (BW), and body weight gain (BWG) as the FI-to-BWG ratio were measured according to the methods reported by Alagawany et al. [16]. At week 13, five rabbits/group were weighed and killed to evaluate the carcass characteristics. All carcass parameters (the main body, liver, head, kidneys, lungs, heart, and other total edible parts) and dressing percentage were measured [17]. 

### 2.5. Digestibility Trial

At week 13, four rabbits from each group were randomly selected and individually distributed into metabolic cages for seven days for the digestibility trial. The feces were collected daily from each rabbit and weighed, and a 50% subsample was obtained, oven-dried at 60 °C for 24 h, and stored in the lab for chemical analysis. Samples of the diets and feces were chemically analyzed [11]. Total digestible nutrients (TDN) were calculated using the following formula [18]:

TDN (%) = DCP (%) + DCF (%) + DNFE (%) + (DEE (%) × 2.25).

The following equation of Schieman et al. [19] was used to calculate the digestible energy (DE) values (kcal/kg diet) of the diets:

DE = 5.28 DCP + 9.51 DEE + 4.2 DCF+ 4.2 DNFE

where DCP is the digestible CP (g), DEE is the digestible EE (g), DCF is the digestible CF (g), and DNFE is the digestible NFE (g). 

### 2.6. Blood Biochemistry

Blood samples were collected from the sacrificed rabbits (five rabbits/group) in sterile tubes. The samples were coagulated and centrifuged (4000 rpm for 10 min), and the sera were stored till further analysis. The serum samples were used to estimate the level of albumin (g/dl), total protein (g/dl), urea (mg/dl), creatinine (mg/dl), total cholesterol (mg/dl), triglyceride (mg/dl), low-density lipoprotein (LDL) (mg/dl), and high-density lipoprotein (HDL) by using kits and a spectrophotometer (Shimadzu, Japan).

### 2.7. Microbiological Analysis

The dietary samples (25 g of each diet) of alfalfa hay, PLM, and their mixture (50/50) were subjected to microbiological analysis at the end of the experimental period. Microbial counts (total bacterial count, *Enterococcus*, coliforms, and total fungi) were estimated using the methods of Xia et al. [20]. For microbial enumeration in the cecum, at week 13, five rabbits per group were chosen and killed. The samples (1–2 g/rabbit) of fresh cecal digesta were subjected to a stream of CO_2_ in bottles and transferred immediately to the laboratory for microbiological investigation. The microbial counts (total bacterial count, *Enterococcus*, coliforms, and *Clostridium*) were determined according to the methods of Xia et al. [20]. 

### 2.8. Statistical Analysis

All statistical analyses were performed using SAS [21]. The data for performance, carcass traits, blood chemistry, and microbiological parameters were analyzed with one-way ANOVA by using the post hoc Newman-Keuls test (*p* < 0.05).

## 3. Results and Discussion

### 3.1. Amino Acid Profile and Mineral Content

Table 2 shows the amino acid compositions of PLM and alfalfa hay. The contents of tyrosine, methionine, and histidine were 3.6%, 3.0%, and 4.8% of CP in PLM and 3.1%, 1.0%, and 1.6% of CP in alfalfa hay, respectively. The contents of lysine, leucine, and isoleucine were 3.8%, 4.6%, and 4.0% of CP in PLM and 5.8%, 7.3%, and 4.7% of CP in alfalfa hay, respectively. The results revealed that threonine, phenylalanine, valine, cysteine, arginine, and total amino acids contents were nearly similar in PLM and alfalfa hay. The contents of all amino acids were similar in all the diets containing PLM and alfalfa hay. 

The results obtained by Guoqiang and Jianqing [23] showed that the contents of threonine, cystine, valine, methionine, leucine, phenylalanine, lysine, histidine, and arginine in healthy paulownia leaves were 0.98%, 0.16%, 0.36%, 0.46%, 0.33%, 0.75%, 0.82%, 0.26%, and 0.85%, respectively. The findings of our study are partially similar to those of Guoqiang and Jianqing [23], especially with respect to the contents of methionine, phenylalanine, and arginine.

The mineral composition of PLM, alfalfa hay, and experimental diets is shown in Table 2. The analyses showed that paulownia has 0.36%, 0.16%, 0.07%, 0.28%, 7.27 mg/kg, and 2.29 calcium, phosphorus, iron, magnesium, copper, and Ca/P, respectively. The value of manganese was 52.63 mg/kg in PLM and 30.67 mg/kg in alfalfa hay. The zinc content of PLM (132.92 mg/kg) was very high when compared with that of alfalfa hay (17.07 mg/kg). The mineral composition in all diets was nearly similar, except for zinc content (47.22, 63.97, and 80.73 mg/kg in the control, 15%, and 30% PLM diets, respectively). According to El-Showk and El-Showk [8], PLM is rich in minerals such as calcium (2.1%), phosphorus (0.6%), zinc (0.9%), and iron (0.6%). Moreover, the levels of heavy metals such as cadmium and lead in the paulownia leaves did not exceed noxious levels [24].

### 3.2. Chemical Composition of PLM

When PLM is used in a complete rabbit diet, the nutrient composition of PLM is considered and mixed with appropriate feed ingredients to obtain a suitable diet formula that meets the nutritional requirements of rabbits. The nutrient composition of PLM, alfalfa hay, and experimental diets is shown in Table 2. The analyses indicated that the contents of CF, NDF, and ash were 23.87%, 40.47%, and 8.85% in PLM and 25.68%, 42.07%, and 11.67% in alfalfa hay, respectively. The content of EE was 3.84% in PLM and 2.33% in alfalfa hay. The CP content was nearly similar in PLM and alfalfa hay. Our findings are partially consistent with those of Stewart et al. [4], who found that the NDF, CP, ash, and fat contents were 29%–55%, 14%–23%, 6%–9%, and 2%–4%, respectively, indicating that paulownia leaves have the potential to be a new feed ingredient for livestock. According to the analysis of El-Showk and El-Showk [8], paulownia leaves contain 22.6% CP and 7.8% ash. Moreover, according to the analysis of Koleva et al. [10] and Bodnár et al. [25], *Paulownia elongata* leaves can be used as a new feed ingredient in diets for ruminants and other herbivorous agricultural species such as rabbits and horses. In the present study, the content of all nutrients was nearly similar in the experimental diets containing PLM and alfalfa hay. *Paulownia* species such as *P. fortunei, P. tomentosa*, and *P. elongata* are palatable and have a good chemical composition and nutritional value suitable for animal feed [26]. 

### 3.3. Growth Performance and Carcass Traits

The effects of dietary treatments on the performance of rabbits are illustrated in Table 3. Generally, all performance parameters were not significantly influenced by dietary paulownia inclusion in the growing rabbit diets, apart from BWG, FI, and feed conversion ratio (FCR) at 5–9 weeks of age. 

Initially, at 5–9 weeks of age, BWG was significantly (*p* = 0.027) decreased because of 30% paulownia when compared with 30% alfalfa hay. The lowest FI value (*p* = 0.003) was detected in the rabbits fed the diet containing 15% PLM. The best FCR value was observed in the rabbits fed 15% PLM during 5–9 weeks of age, when compared with 30% PLM (*p* = 0.001). The decrease in BWG (with 30% PLM) and FI (with 15% PLM) during the first 4 weeks of the experiment (5–9 weeks of age) might be due to the slight bitterness of the PLM possibly affecting palatability, some pharmacologically active agents, or the organic matter (OM) being simply less digestible. Leaves of paulownia have been reported to be slightly bitter but palatable for some animal species such as sheep and cattle [8]. Also, in the current study, there was a slight decrease in the digestion of organic matter in the 30% PLM group, which could reduce the BWG of the rabbits during the period of 5–9 weeks of age.

During the overall experimental period, 5–13 weeks of age, there were no significant differences in the BWG, FI, or feed utilization among the experimental groups. Previous studies have reported many fodder trees and shrubs that may partially or completely replace concentrated rations without any detrimental effects on the digestion or growth of animals such as goats and sheep [27]. Also, Wang and Shogren [28] and Zhao-Hua et al. [29] have confirmed that paulownia leaves can be used in the feeds for pigs and rabbits without any negative effects, which is consistent with our results. In China, Barton et al. [30] reported that pellets with up to 20% paulownia leaves are manufactured for fish and chicken feed. Moreover, dietary supplementation with up to 10% tree leaf meal had nonsignificant impacts on FI, BWG, and FCR [31].

In the present study, all carcass traits (carcass yield, heart, kidneys, spleen, lungs, giblets, and dressing percentages) were not (*p >* 0.05) statistically influenced by the PLM treatments (Table 4). An adequate number of dietary tree leaves could have a positive impact on the growth and carcass parameters of animals. Better feed utilization might be due to antimicrobial properties against several pathogenic microbes [32]. Abubakar et al. [33] stated that growing rabbits can use varying dietary levels of tree leaves up to 45% without any adverse impacts on the growth rate and organ and carcass characteristics. Adedeji et al. [34] found that the inclusion of 5%, 10%, and 15% tree leaves improved the performance of rabbits, although the feed consumption decreased.

### 3.4. Nutrient Digestibility and Nutritive Value

The effects of dietary inclusion of PLM on DM, CP, CF, OM, EE, and NFE digestibility and TDN, DCP, and DE values of growing rabbits are shown in Table 5. No significant differences (*p* > 0.05) were observed in the nutrient digestibility, apart from NFE, because of the paulownia treatments. Inclusion of paulownia in the rabbit diets significantly reduced (*p* = 0.028) NFE digestibility. As a result, decreased TDN and DE were observed when the animals were fed 30% paulownia foliage. Conversely, the inclusion of 15% paulownia foliage, when compared with alfalfa hay, in the rabbit rations did not affect TDN and DE. Our results are consistent with those reported by Juniar et al. [31], who postulated that low levels of tree leaf meal have no adverse impacts on the digestion coefficient and even improve the feed conversion ratio. El-Gendy [35] observed that up to 30% *Acacia saligna* leaf meal did not affect nutrient digestibility and N balance. Tree leaves can be used as a supplement to enhance feed utilization and animal performance or to replace traditional feeds to achieve safe and economic production [9,36]. Rubanza et al. [37] found that nutrient digestibility was improved in animals fed tree leaves, and this improvement may be due to the high nutritional profile, especially CP, EE, NDF, acid detergent fiber (ADF), gross energy (GE), and amino acids. However, a berseem hay diet decreased the digestibility of organic matter (*p* = 0.028), dry matter (*p* = 0.004), crude protein (*p* = 0.001), acid detergent fiber (*p* = 0.011), and NDF (*p* = 0.033); in addition, TDN (*p* = 0.045) and DCP (*p* = 0.041) were detected in the diets containing tree leaves [5]. 

To the best of our knowledge, the fibrous ingredients such as alfalfa hay and tree leaves used in the present study have not been examined previously, and, in this study, a high amount of this feed ingredient (up to 30%) was used. The digestibility of DM, OM, EE, CP, and CF can be assumed to be within the range of values reported in rabbits.

### 3.5. Blood Biochemistry

Blood biochemical analysis is a defined way to evaluate the clinical and health status of animals and humans [38]. The effects of dietary inclusion of paulownia on blood parameters such as albumin, urea, total protein, creatinine, total cholesterol, triglyceride, HDL, and LDL are shown in Table 6. All blood parameters were not significantly (*p* > 0.05) affected by the dietary treatments, except HDL and LDL. Our results were within the normal levels reported by Mader [39]. Similarly, no significant impacts on most serum parameters were detected in rabbits fed rations containing 0%, 5%, 10%, and 15% tree foliage, which is consistent with our results [40]. 

In contrast, Varlyakov et al. [41] found that blood parameters were significantly affected by the dietary inclusion of paulownia in sheep diets. PLM increased the serum levels of total protein, albumin, and globulin. Furthermore, they showed that paulownia leaves have the ability to significantly reduce blood glucose levels [41]. This discrepancy might be related to the differences in animal species or PLM inclusion level. On the other hand, herein, the HDL level was significantly increased in the rabbits that received the PLM diets (*p* = 0.037), but LDL was decreased (*p* = 0.042) in those that received 30% PLM. Our findings are partially consistent with those of Sun et al. [42], who detected that the inclusion of 10% tree foliage in rabbit diets resulted in the lowest value of serum LDL when compared with the alfalfa group. These effects could be linked to the PLM antioxidant and antiperoxide activity [4] or the reduction of energy intake [43]. Our results suggested PLM dietary inclusion had no negative effect on blood biochemistry, so it could be used safely up to 30%.

### 3.6. Microbial Populations

Rabbits have a special characteristic (cecotrophy) that helps them to use a high-roughage diet efficiently. The microflora in the cecum help in the digestion of the fibrous ingredients. 

The microbial populations in the digestive system of ruminants affect the food before it reaches the main place of absorption and digestion, but the microbial populations in the cecum of rabbits affect the undigested residues of feed. Tree leaves could be fed to rabbits separately as green fodder [44]. As shown in Table 7, the total fungi, Enterobacteriaceae, and coliform counts and total bacterial count in all the dietary treatments were varied (*p* < 0.05). The populations of total fungi and Enterobacteriaceae and total bacterial count were decreased (*p* < 0.05) in all the paulownia groups when compared with the control. However, the coliform count increased in the 30% PLM group, followed by that in the control (0% PLM) and 15% PLM groups. In the cecum, inclusion of paulownia in the rabbit diets had a positive effect on the microbial populations (Table 8). The populations of coliform and Enterobacteriaceae and total bacterial count decreased (*p* ≤ 0.001) in the rabbits fed the paulownia diets. However, inclusion of paulownia in the rabbit diets did not affect (*p* = 0.131) cecal *Clostridium* populations. The inclusion of 15% and 30% paulownia delayed the growth of fungi and bacteria. 

The results indicated that the inclusion of paulownia in the rabbit diets resulted in a delay in spoilage emergence, which may be attributed to the antimicrobial effects of paulownia because of its biochemical composition [45]. Flavonoids extracted from paulownia leaves have antiradical activity and cell-protective effects [46]. An in vitro study showed that aqueous extracts of fresh paulownia leaves have antimicrobial effects against *Pseudomonas aeruginosa, Salmonella entericа, Streptococcus pyogenes, Staphylococcus aureus, Candida albicans,* and *Paenibacillus alvei.* The inhibitory impact of paulownia leaves is more evident against Gram-negative bacteria [47]. Ibrahim et al. [48] showed that the constituents of paulownia oil have antimicrobial effects and could be used as a good source of pharmaceutical products.

## 4. Conclusions

Our study provides evidence for the suitability of using PLM as a feed resource for animals. Throughout the experiment period, the inclusion of 15% PLM in the rabbit diet did not adversely affect growth performance, blood biochemistry, carcass traits, nutrient digestibility, and nutritional values. However, the inclusion of 30% PLM significantly reduced the digestible energy and NFE digestibility; nonsignificantly decreased growth performance; and did not negatively affect blood measurements, carcass characteristics, and digestibility of most nutrients. Therefore, the current study recommends the inclusion of 15% PLM in rabbit diets instead of alfalfa hay to achieve the maximum benefit. In addition, the populations of fungi and Enterobacteriaceae and total bacterial count in the feed, and populations of coliforms and Enterobacteriaceae and total bacterial count in the cecum content, were decreased in the rabbits fed diets containing PLM.

## Figures and Tables

**Table 1:** Ingredients (percentage of the as-fed diet) of the growing rabbit diets.

Items	Paulownia Leaf Meal (%)		
	Control (0%)	15%	30%
Alfalfa hay	30	15	0
Paulownia leaf meal	0	15	30
Soybean meal	15	15	15
Wheat bran	24.8	24.5	24
Barely grain	28	28	28
Limestone	1.2	1.5	2
NaCl	0.5	0.5	0.5
Premix *	0.5	0.5	0.5
Total	100	100	100

* Each kilogram of premix (vitamin mixture) contained vitamin A, 20,000 IU; vitamin D3, 15,000 IU; vitamin E, 8.33 g; vitamin K, 0.33 g; vitamin B1, 0.33; vitamin B2, 1.0 g; vitamin B6, 0.33 g; vitamin B5, 8.33 g; vitamin B12, 1.7 mg; pantothenic acid, 3.33 g; biotin, 33 mg; folic acid, 0.83 g; and choline chloride, 200 g.

**Table 2:** Nutrient composition of paulownia leaf meal, alfalfa hay, and experimental diets.

Items	Paulownia Leaf Meal	Alfalfa Hay	Experimental Diets		
			Control (30% hay)	15% Hay + 15% Paulownia	30% Paulownia
Chemical composition (% on DM basis)					
Crude protein	17.41	17.29	18.47	18.63	18.65
Ether extract	3.84	2.33	2.45	2.67	2.87
Crude fiber	23.87	25.68	13.18	12.87	12.54
Neutral detergent fiber	40.47	42.07	34.04	33.96	33.79
Dry matter	88.12	89.65	89.94	89.74	89.36
Ash	8.85	11.67	9.65	8.92	8.76
Nitrogen free extract	46.03	43.03	56.25	56.91	57.18
Amino acid composition (% of crude protein, N× 6.25) ^1^					
Essential amino acids					
Threonine	4.2	4.1	3.88	3.88	3.91
Cysteine	1.9	2.1	1.90	1.86	1.85
Valine	4.3	4.7	5.33	5.26	5.23
Methionine	3.0	1.0	1.30	1.58	1.87
Isoleucine	4.0	4.7	5.05	4.93	4.86
Leucine	4.6	7.3	7.55	7.14	6.79
Tyrosine	3.6	3.1	2.58	2.64	2.73
Phenylalanine	4.4	4.6	4.88	4.83	4.82
Histidine	4.8	1.6	2.34	2.79	3.25
Lysine	3.8	5.8	5.51	5.21	4.96
Total	38.6	39.0	40.31	40.13	40.28
Nonessential amino acids					
Arginine	5.2	5.2	6.56	6.53	6.56
Asx ^2^	6.8	11.4	NC	NC	NC
Proline	13.6	5.8	NC	NC	NC
Glx ^2^	7.6	9.3	NC	NC	NC
Serine	4.6	4.1	NC	NC	NC
Glycine	6.3	4.4	NC	NC	NC
Alanine	3.7	5.2	NC	NC	NC
Total	47.8	45.4	NC	NC	NC
Total amino acids	86.4	84.4	NC	NC	NC
Nonprotein nitrogen	13.6	15.6	NC	NC	NC
Mineral composition (on DM basis)					
Calcium (%)	0.36	1.48	1.05	1.00	0.94
Phosphorus (%)	0.16	0.26	0.63	0.61	0.59
Iron (%)	0.07	0.02	0.01	0.02	0.03
Magnesium (%)	0.28	0.32	0.32	0.31	0.30
Manganese (mg/kg)	52.63	30.67	49.44	52.35	55.11
Copper (mg/kg)	7.27	21.53	17.03	14.88	12.70
Zinc (mg/kg)	132.92	17.07	47.22	63.97	80.73
Ca/P	2.29	5.78	1.65	1.64	1.58

^1^ The content of the three experimental diets of amino acids was calculated according to the Nutrient Requirements of Rabbits (NRC) [22] and the analyzed composition of paulownia leaf meal and alfalfa hay. NC= not calculated. ^2^ Asx and Glx represent the sum of asparagine and aspartic acid, and glutamine and glutamic acid, respectively.

**Table 3:** Effects of paulownia leaf meal on the performance of New Zealand White rabbits during the fattening period.

Item	Paulownia Leaf Meal (%)			SEM ^1^	*p*-Value ^2^
	Control (0%)	15%	30%		
Live body weight (g) at					
Week 5	541.22	529.33	523.11	16.85	0.912
Week 9	1401.46	1232.13	1092.53	59.21	0.108
Week 13	2096.80	1973.46	1875.20	88.89	0.607
Cumulative body weight gain (g) at					
Weeks 5–9	860.24 ^a^	722.35 ^ab^	574.44 ^b^	42.80	0.027
Weeks 9–13	695.33	741.33	777.20	40.58	0.695
Weeks 5–13	1555.57	1463.68	1351.64	71.85	0.529
Feed intake (g) at					
Weeks 5–9	78.38 ^a^	62.31 ^b^	84.55 ^a^	2.51	0.003
Weeks 9–13	125.25	118.42	123.46	4.06	0.816
Weeks 5–13	101.81	90.27	104.01	2.92	0.126
Feed:gain ratio (g/g) at					
Weeks 5–9	2.82 ^b^	2.44 ^b^	4.31 ^a^	0.21	0.001
Weeks 9–13	5.23	4.83	4.79	0.31	0.820
Weeks 5–13	4.03	3.63	4.55	0.22	0.245

Different superscripts within the same row are significantly different (*p* < 0.05). ^1^ SEM = pooled standard error of means. ^2^ Overall treatment *p*-value.

**Table 4:** Effects of paulownia leaf meal on carcass traits of growing New Zealand White rabbits at 13 weeks of age.

Item	Paulownia Leaf Meal (%)			SEM ^1^	*p*-Value ^2^
	Control (0%)	15%	30%		
Carcass yield, %	52.23	50.82	47.98	0.81	0.072
Heart, g/kg SW	2.73	3.16	3.12	0.11	0.267
Kidneys, g/kg SW	9.46	11.37	11.28	0.52	0.279
Liver, g/kg SW	37.21	50.90	46.24	3.01	0.194
Spleen, g/kg SW	0.57	0.49	0.60	0.03	0.418
Lungs, g/kg SW	11.26	7.63	6.21	1.14	0.183
Giblets, %	6.12	7.35	6.74	0.25	0.126
Dressing, %	58.35	58.18	54.72	0.84	0.132

^1^ SEM = pooled standard error of means. ^2^ Overall treatment *p*-value.

**Table 5:** Effects of the tested diets on apparent nutrient digestibility and nutritive values of growing New Zealand White rabbits.

Item	Paulownia Leaf Meal (%)			SEM ^1^	*p*-Value ^2^
	Control (0%)	15%	30%		
Nutrient digestibility (g/kg)					
Dry matter	675.3	677.0	636.3	8.94	0.102
Ether extract	661.1	641.8	611.6	15.10	0.434
Crude protein	768.7	771.8	740.5	9.66	0.373
Crude fiber	397.8	421.0	373.4	13.32	0.372
NFE	749.7 ^a^	731.9 ^ab^	698.6 ^b^	8.46	0.028
Organic matter	699.0	697.7	657.4	8.93	0.087
Nutritive values					
DCP (g/kg)	143.2	143.9	136.8	1.86	0.234
TDN (g/kg)	660.8 ^a^	656.7 ^a^	612.7 ^b^	9.02	0.040
DE (kcal/kg diet)	2931 ^a^	2915 ^a^	2722 ^b^	39.7	0.043

Different superscripts within the same row are significantly different (*p* < 0.05). ^1^ SEM = pooled standard error of means. ^2^ Overall treatment *p*-value; NFE, nitrogen-free extract; DCP, digestible crude protein; TDN, total digestible nutrients; DE, digestible energy.

**Table 6:** Effects of paulownia leaf meal on the blood chemistry of growing New Zealand White rabbits at 13 weeks of age.

Item	Paulownia Leaf Meal (%)			SEM ^1^	*p*-Value ^2^
	Control (0%)	15%	30%		
Total protein (g/dl)	7.23	8.49	9.60	0.43	0.061
Albumin (g/dl)	3.17	3.06	2.97	0.21	0.570
Urea (mg/dl)	18.14	18.14	20.39	1.27	0.764
Creatinine (mg/dl)	1.04	1.05	1.11	0.08	0.959
Total cholesterol (mg/dl)	68.96	72.06	67.80	3.39	0.899
Triglyceride (mg/dl)	98.72	107.05	103.40	3.61	0.702
HDL (mg/dl) ^3^	30.10 ^b^	42.05 ^a^	44.87 ^a^	2.77	0.037
LDL (mg/dl) ^3^	44.54 ^a^	40.67 ^a^	32.61 ^b^	2.17	0.042

Different superscripts within the same row are significantly different (*p* < 0.05). ^1^ SEM = pooled standard error of means. ^2^ Overall treatment *p*-value; ^3^ HDL: high-density lipoprotein; LDL: low-density lipoprotein.

**Table 7:** Effects of paulownia leaf meal on total bacterial count and *Enterococcus*, coliform, and total fungi populations in the basal diet during feeding of growing New Zealand White rabbits.

Item	Paulownia Leaf Meal (%)			SEM ^1^	*p*-Value ^2^
	Control (0%)	15%	30%		
Microbiological count (Log CFU/g)					
Total bacterial count	4.53 ^a^	4.39 ^b^	4.37 ^b^	0.02	0.021
*Enterococcus*	2.68 ^a^	1.88 ^c^	2.50 ^b^	0.12	<0.001
Coliforms	2.28 ^b^	1.80 ^c^	2.41 ^a^	0.09	<0.001
Total fungi	2.32 ^a^	1.01 ^b^	1.01 ^b^	0.22	<0.001

Different superscripts within the same row are significantly different (*p* < 0.05). ^1^ SEM = pooled standard error of means. ^2^ Overall treatment *p*-value.

**Table 8:** Effects of paulownia leaf meal on cecal microbiota (total bacterial count and *Enterococcus*, coliform, and *Clostridium* counts) in growing New Zealand White rabbits.

Item	Paulownia Leaf Meal (%)			SEM ^1^	*p*-Value ^2^
	Control (0%)	15%	30%		
Microbiological count (Log CFU/g)					
Total bacterial count	8.56 ^a^	8.45 ^b^	8.45 ^b^	0.01	0.001
*Enterococcus*	6.46 ^a^	6.05 ^c^	6.18 ^b^	0.06	<0.001
Coliforms	5.55 ^a^	5.43 ^b^	5.43 ^b^	0.01	<0.001
*Clostridium*	4.58	4.84	4.14	0.14	0.131

Different superscripts within the same row are significantly different (*p* < 0.05). ^1^ SEM = pooled standard error of means. ^2^ Overall treatment *p*-value.
